# Deep Phosphoproteomic Measurements Pinpointing Drug Induced Protective Mechanisms in Neuronal Cells

**DOI:** 10.3389/fphys.2016.00635

**Published:** 2016-12-23

**Authors:** Chengli Yu, Jing Gao, Yanting Zhou, Xiangling Chen, Ruoxuan Xiao, Jing Zheng, Yansheng Liu, Hu Zhou

**Affiliations:** ^1^Department of Analytical Chemistry and CAS Key Laboratory of Receptor Research, Shanghai Institute of Materia Medica, Chinese Academy of SciencesShanghai, China; ^2^College of Pharmacy, University of Chinese Academy of Sciences, Chinese Academy of SciencesBeijing, China; ^3^Shanghai Key Laboratory of New Drug Design, School of Pharmacy, East China University of Science and TechnologyShanghai, China; ^4^Department of Biology, Institute of Molecular Systems Biology, Swiss Federal Institute of Technology in ZurichZurich, Switzerland

**Keywords:** phosphoproteomics, Alzheimer's disease, drug effect, mass spectrometry, neutronal cells

## Abstract

Alzheimer's disease (AD) is a progressive and irreversible neurological disorder that impairs the living quality of old population and even life spans. New compounds have shown potential inneuroprotective effects in AD, such as GFKP-19, a 2-pyrrolidone derivative which has been proved to enhance the memory of dysmnesia mouse. The molecular mechanisms remain to be established for these drug candidates. Large-scale phosphoproteomic approach has been evolved rapidly in the last several years, which holds the potential to provide a useful toolkit to understand cellular signaling underlying drug effects. To establish and test such a method, we accurately analyzed the deep quantitative phosphoproteome of the neuro-2a cells treated with and without GFKP-19 using triple SILAC labeling. A total of 14,761 Class I phosphosites were quantified between controls, damaged, and protected conditions using the high resolution mass spectrometry, with a decent inter-mass spectrometer reproducibility for even subtle regulatory events. Our data suggests that GFKP-19 can reverse Aβ_25−35_ induced phosphorylation change in neuro-2a cells, and might protect the neuron system in two ways: firstly, it may decrease oxidative damage and inflammation induced by NO via down regulating the phosphorylation of nitric oxide synthase NOS1 at S847; Secondly, it may decrease tau protein phosphorylation through down-regulating the phosphorylation level of MAPK14 at T180. All mass spectrometry data are available via ProteomeXchange with identifier PXD005312.

## Introduction

Alzheimer's disease (AD) is a progressive and irreversible neurological disorder characterized by memory and cognitive impairment. AD is currently involved in 50–70% of dementia cases with half of people over 85 years of age suffering from it (Anand et al., 2014; Imtiaz et al., 2014). It is estimated that the prevalence of AD in year of 2050 would reach 88 million cases worldwide (Van Cauwenberghe and Van Broeckhoven, 2016).

Among the medicines approved by the U.S. Food and Drug Administration (FDA), Donepezil, Galantamine, Reivastigmine, and Tacrine, for instance, are cholinesterase inhibitors dealing with the loss of cholinergic neurons (Amemori et al., 2015), while memantine has the effect to block the cytotoxicity of excess extracellular glutamate on neurons as an N-Methyl-D-aspartate receptor (NMDAR) antagonist (Arbor et al., 2016). It seems that most of the current therapeutic drugs for AD are designed to provide symptomatic treatment rather than to restore the declined brain function.

The two main neuropathological characteristics of AD have been recognized, namely senile plaques (SPs) and neurofibrillary tangles (NFTs), which are, respectively, associated to amyloid β (Aβ) peptides and hyperphosphorylated tau protein (Holtzman et al., 2011; Marsden et al., 2011). With important functional consequences in neurogenesis, amyloid precursor protein (APP) could be cleaved by β-secretase at the N-terminal of an Aβ sequence, and then cleaved by γ-secretase subsequently to produce an Aβ fragment (De Strooper, 2010). Among these key molecules, Aβ is a peptide with 40~42 amino acids most commonly in length, aggregation of which could lead to NFTs, vascular damage, neuronal loss, and even dementia (Hardy and Higgins, 1992). Tau is a microtubule-associated protein (MAP) necessary for stabilizing microtubules, and proper phosphorylation of tau within the microtubules is required for neurite outgrowth (Lindwall and Cole, 1984; Caceres and Kosik, 1990). Nevertheless, hyperphosphorylated tau has been validated in AD, which is able to further promote its dissociation from microtubules and thus resulting in protein destabilization and disruption of neuronal transport (Garcia and Cleveland, 2001). Moreover, mitochondrial dysfunction has been found in multiple AD tissues (Swerdlow, 2002). Accordingly the existing mitochondrial cascade hypothesis assumes that it is the brain mitochondrial dysfunction in AD that induce amyloidosis and tau phosphorylation (Swerdlow and Khan, 2004). Furthermore, memory impairment and dementia are common symptoms of AD. Significant cholinergic dysfunctions in central nervous system of AD had been found. These studies, combined with the emerging role of cholinergic system (Drachman and Leavitt, 1974), led to cholinergic hypothesis which proposes that cognitive disorder in patients with AD is dramatically associate to the loss of cholinergic neurons in the basal forebrain (Bartus et al., 1982). Based on the above mechanisms, acetylcholinesterase (AChE) inhibitors, such as donepezil, galantamine, and rivastigmine and NMDAR antagonist like memantine have been common medicines for AD. Considering inflammation and oxidative damage are reported in AD (Galimberti and Scarpini, 2011) and anti-inflammatory drugs and antioxidants have been made to slow the progression of AD (Cole and Frautschy, 2010; Lee et al., 2010).

With its high sensitivity, high accuracy and high selectivity recently achieved, mass spectrometry (MS) increasingly plays a vital role in biomarker discovery, pharmaceutical research, and in revealing molecular mechanisms of drug actions and activities (Aebersold and Mann, 2003, 2016). In the research field of AD, for example, Lynn et al. utilized a 2-dimensional liquid chromatography-tandem mass spectrometry (LC-MS/MS) and the isotope coded affinity tag (ICAT) method to identify, quantify and distinguish potentially interacting mitochondrial proteins in short postmortem temporal pole specimens with mild cognitive impairment, early AD, late-stage AD and age-matched normal control subjects (Lynn et al., 2010). Similarly, an LC-MS/MS based approach was developed by Hejazi et al to investigate the content of sphingomyelin, ceramide, and galactosylceramide in hippocampus from AD and control subjects, which could be further applied for lipid analysis (Hejazi et al., 2011). Also, Dillen et al. set up a screening UHPLC–MS/MS method for analysis of amyloid peptides in cerebrospinal fluid, thus laying foundation for the preclinical study (Dillen et al., 2011). Currently, despite the development of label-free proteomic methods, the isotopic labeling methods still present the most accurate quantification approach, especially for those regulations with a small fold change (FC) (Walther et al., 2015). Stable isotope labeling by amino acids in cell culture (SILAC) (Ong et al., 2002) is a powerful metabolic labeling strategy in MS-based quantitative proteomics, when combined with phosphoproteomics can provide efficient and robust help to test quantitative changes in phosphorylation sites (Dengjel et al., 2007; Dephoure et al., 2008; Hilger et al., 2009). The combination of phosphoproteomics and SILAC labeling is therefore very promising to be tested for analyzing post-translational regulations in AD related research areas.

As an example, we selected GFKP-19, which is a 2-pyrrolidone derivative compound, and could be a promising drug candidate for AD treatment. The pyrrolidone derivative racetam drugs, such as aniracetam is an ampakine nootropic with glutamatergic activity and neuroprotective potential (Koliaki et al., 2012). The 2-pyrrolidone clausenamideare is an anti-dementia drug candidate and been reported to inhibit the cytotoxicity of beta amyloid, tau hyperphosphorylation and neurodegeneration (Hu et al., 2010; Chu and Zhang, 2014; Chu et al., 2016). To mimic the injury of neuron cells in AD, we used the active Aβ_25−35_ peptide (peptide containing the 25–35 th amino acids of Aβ). Aβ_25−35_ peptide exhibits the similar cytotoxic properties of the full-length protein Aβ_1−42_, and has been reported to be the active fragment of Aβ_1−42_ (Pike et al., 1995; Clementi et al., 2005). In previous studies, Aβ_25−35_ treatment can induce apoptosis in many mammalian cell lines *in vitro*. To determine if GFKP-19 can remit Aβ_25−35_ induced effect and the mechanisms underlying its neuroprotective effects, we carried out a phosphoproteomic study measuring the cellular signaling changes in different states of neuro-2a cell by DMSO, Aβ_25−35_ alone or Aβ_25−35_ together with GFKP-19 treatment. The phosphorylation of tau increased after Aβ_25−35_ treatment, and the increase can be reversed by GFKP-19. Interestingly, the changes in the phosphorylation of tau kinases also increased during Aβ_25−35_ treatment and decreased with the addition of GFKP-19. The comprehensive profiling of phosphorylation regulation events by SILAC and MS provides a general framework for understanding drug actions, which is for the first time reported for the AD relevant drug candidate compounds.

## Materials and methods

### Materials

Aβ_25−35_ was purchased from Sigma (Sigma, A4559). N2 supplement was purchased from Gibco (Gibco, 17502048). As an example of 2-pyrrolidone derivative compounds GFKP-19 was kindly provided by a collaboration laboratory, which wishes to not release the detailed structure of this compound along with the present paper. Aβ_25−35_ was dissolved in DMSO at a concentration of 3 mM. GFKP-19 was dissolved in DMSO at a concentration of 10 mM. 3-(4, 5-dimethylthiazolyl-2)-2, 5-diphenyl tetrazolium bromide (MTT) was purchased from MP Biomedicals (MP Biomedicals, 02102227). The dialyzed fetal bovine serum (FBS) was from Hyclone (Hyclone, SH30079.03). The Amicon Ultra 15 mL centrifugal filter with a membrane NMWL of 30 kDa was purchased from Merck Millipore (Merck Millipore, UFC903024).

### Cell culture

Neuro-2a cells purchased from American Type Culture Collection (ATCC, CCL-131) were cultured in DMEM media supplemented with 10% FBS, 1% penicillin/streptomycin, growing in humidified incubator at 37°C and 5% CO_2_. N2 supplement (Gibco, 17502048) was used as a serum-free supplement during drug treatment. Neuro-2a cells were evenly seeded into 96-well plates with a density of 5 × 10^3^ cells per well. At the second day, cells were pretreated with or without GFKP-19 (0.1 or 1 μM) for 1 h, and Aβ_25−35_ (1 μM) was added in the presence of GFKP-19 and all cells were incubated for another 24 h. Control cells were treated with corresponding concentration of DMSO.

### Determination of cell viability

Cell viability was measured by MTT assays (Mosmann, 1983; Ulukaya et al., 2004). After treatments above, cells were incubated with 0.5 mg/mL MTT for 4 h. Then, the supernatant was removed carefully and 150 μL DMSO was added to dissolve the formazan. The plate was shaken for 10 min, and absorbance at 490 nm was obtained with a microplate reader (BIO-RAD Model 3550).

### SILAC labeling and sample preparation

DMEM media for SILAC (Thermo, 89985) was supplemented with 10% dialyzed FBS, 1% penicillin/streptomycin and before drug treatment, and the dialyzed FBS was replaced by N2 supplement during drug treatment. The light SILAC amino acids L-lysine (Sigma, L8662) and L-arginine (Sigma, A8094), the medium SILAC amino acids L-lysine-^2^H_4_ (Sigma, 616192) and L-arginine-^13^C_6_ (Sigma, 643440), or the heavy SILAC amino acids L-lysine-^13^${\text{C}}_{6}^{15}$N_2_ (Sigma, 608041) and L-arginine-^13^${\text{C}}_{6}^{15}$N_4_ (Sigma, 608033) were added with a final concentration of 28 mg/L for the arginine and 50 mg/L for the lysine amino acids to generate light, medium, and heavy SILAC-labeling media. Neuro-2a cells were labeled as previously published with light, medium and heavy SILAC-labeling media (Ong and Mann, 2006). After eight passages, full incorporation of SILAC amino acid was confirmed.

Light SILAC-labeling cells were taken as controls, whereas medium SILAC-labeling cells were treated with Aβ_25−35_ (1 μM) for 4 or 24 h, and heavy SILAC-labeling cells were pretreated with GFKP-19 for 1 h followed exposure to Aβ_25−35_ (1 μM) and GFKP-19 for another 4 or 24 h. All cells were, respectively, lysed in buffer containing 4% SDS, 0.1M DTT, 100 mM tris (pH 7.5). The protein concentrations were evaluated by tryptophan fluorescence emission assay (Kulak et al., 2014) and evaluated by SDS-polyacrylamide gel electrophresis (SDS-PAGE). 100 ng/μL tryptophan was used to build a standard calibration curve (0, 0.125, 0.25, 0.5, 0.75, 100, and 150 μL), and 1 μL of sample was diluted in 200 μL of 8 M urea for reading protein amount. An excitation wavelength of 295 nm was adopted to determine protein concentrations whereby tryptophan fluorescence emission at 350 nm. The assaying instrument used was SpectraMax M5 (Molecular Devices, Sunnyvale, CA). Protein extracts derived from different treatments were then mixed at the ratio of 1:1:1 at the protein level. A total of 15 mg protein mixture was alkylated with iodoacetamide and digested by trypsin following the filiter-aided sample preparation method (FASP) reported (Wisniewski et al., 2009). The enzymatic hydrolysis efficiency was around 40~50%. The peptides were subjected to TiO_2_ enrichment to get phosphopeptides as described below.

### TiO_2_ enrichment of phosphopeptides

Phosphopeptides were enriched by using Titansphere-chromatography (TiO_2_) as described (Thingholm et al., 2006; Selenica et al., 2007) with minor modifications. Briefly, 1 mg of slurry of 5 μm Titansphere material (GL Sciences) in 80% ACN/1% TFA containing saturated glutamic acid was added directly to the 1 mg tryptic peptides. The slurry was incubated for 15–30 min at room temperature with end-over-end rotation, spunbriefly on a bench-top centrifuge, and the supernatant was collected. Titansphere material with phosphopeptides bounded was washed two times with 400 μL 30% ACN/0.5% TFA and 2 times with 400 μL 70% ACN/0.1% TFA, then transferred on top of a C8 disc (Empore) placed in 200 μL pipette-tip. Bound phosphopeptides were eluted by passing 1 times 50 μL 0.6% NH_4_OH/20% ACN and 2 times 50 μL 1% NH_4_OH/40% ACN through the beads. Elutes were then dried almost to completeness. Normally the yield is 1% of the original peptides input. All phosphopeptides got at this step were subjected to fractionation.

### Phosphopeptide fractionation by high-pH reversed-phase LC

Samples were separated by high-pH reversed-phase high-performance liquid chromatography (HPLC). The fractionation procedure was modified from previously described protocol (Batth et al., 2014). A Waters Xbridge BEH C18 2.5 μm 3.0 × 100 mm XP column and an Agilent 1290 HPLC were used. Mobile phase A: 10 mM ammonium formate, 100% H_2_O (adjusted to pH 10 with ammonium hydroxide); B: 10 mM ammonium formate, 10% H_2_O, 90% ACN (adjusted to pH 10 with ammonium hydroxide). Dried phosphopeptides were dissolved in mobile phase A. The temperature of the column was operated at 45°C. After loaded onto the column, samples were separated using a 66 min gradient at a flow rate of 200 μL/min, as follows: 0 min 5% B; 50 min 25% B; 54 min 60% B; 56 min 70% B; 61 min 70% B; 63 min 5% B; 66 min 5% B. All fractions were collected at 2 min intervals. 28 fractions were collected between 0 and 56 min and were pooled into 14 fractions by combining fractions (1 and 15 were combined as fraction 1; 2 and 16 were combined as fraction 2; 3 and 17 were combined as fraction 3, and so on, until 14 and 28 as fraction 14).

### LC-MS/MS analysis

All LC-MS/MS analysis were performed on an EASY n-LC 1000 system directly connected to a Q Exactive (Thermo Fisher Scientific, San Jose, CA) or a Q Exactive HF (Thermo Fisher Scientific, San Jose, CA) mass spectrometer which equipped with a nanospray ion source. Phosphopeptide fractions were dissolved in solvent A (0.1% formic acid, 100% H_2_O) and loaded onto a reversed-phase column, which was packed in-house with 3 μm C18 ReproSil particles (Dr. Maisch GmbH). The inner diameter of the column was 75 μm. The solvent B was 0.1% formic acid in acetonitrile. Samples were separated with a 2 h gradient at a flow rate of 300 nL/min. The gradient was set up as follows: 0–4 min, 3–4% B; 106 min, 20% B; 114 min, 35% B; 117–120 min, 90% B. Each sample was analyzed twice by Q Exactive or Q Exactive HF, respectively.

For Q Exactive, the instrument parameters were set as: The scan range was between 300 and 1500 m/z and full scan resolution was set at 70,000; fragmentation was performed with the high-energy collisional dissociation (HCD) with a normalized collision energy (NCE) of 28%; one full scan was followed by 15 data-dependent MS/MS scans with a resolution of 17,500 and isolation window of 2.0 m/z.

For Q Exactive HF, the instrument parameters were as follows: The full scan used a mass window of 350–1800 m/z at a target resolution of 60,000; precursor ions were fragmented by HCD with a NCE of 27%; one full scan was followed by 20 data-dependent MS/MS scans with a resolution of 30,000 and isolation window of 1.6 m/z.

The MS parameters are firstly set up according to the vender's instructions and then optimized in house to maximize the analytical power of each machine.

### Data analysis

The raw data files were processed using software MaxQuant (Cox and Mann, 2008) version 1.5.1.0 with an false-discovery rater (FDR) <0.01 at all the levels of proteins, peptides and modifications, to generate peak lists. The MS/MS spectra were searched against the Mouse UniProt FASTA database (July 2016) containing 51418 entries. Enzyme specificity was set to trypsin, allowing for cleavage N-terminal to proline and between aspartic acid and proline. The search included cysteine carbamidomethylation as a fixed modification and N-acetylation of protein, oxidation of methionine and/or phosphorylation of serine, threonine tyrosine residue (STY) as variable modifications. The missed cleavage number allowed was set as no more than 3. The SILAC based quantification of peptides and proteins was also performed by MaxQuant. SILAC ratios were normalized so that the median ratio was 1 to correct unequal sample mixing. Bioinformatics analyses were carried out with Microsoft Excel, GraphPad, Perseus (Tyanova et al., 2016), and R (https://www.r-project.org/) statistical computing software. Functional annotations were made through UniProtKB (http://www.uniprot.org/uniprot/), the Database for Annotation, Visualization and Integrated Discovery (DAVID) (https://david.ncifcrf.gov/), motif-x (Schwartz and Gygi, 2005), and Ingenuity Pathway Analysis (IPA, version 8.5, Ingenuity Systems, Redwood City, CA). Interaction network was made with software Cytoscape (http://www.cytoscape.org) and the physical interaction was provided by the SIGnaling Network Open Resource (SIGNOR) (Perfetto et al., 2016). Categorical annotation was performed in the Perseus software environment (Tyanova et al., 2016), which is a following up analysis tool in MaxQuant workflow. Categorical annotation was supplied in the form of GO biological process, molecular function, and cellular component. Enrichment for these categories was evaluated by Fisher exact test. Multiple hypothesis testing was controlled by using a Benjamini-Hochberg FDR threshold of 0.02. The annotation matrix algorithm was used to compute the difference of any significant protein annotation term from the overall intensity distribution as described (Cox et al., 2012). Motif-X was used to test for significantly overrepresented motifs, with the following parameters: *P-*value threshold ≤ 1.0E-6, with the entire mouse database as background database. The fold increase of motif was visualized with the Pheatmap package in R.

## Results and discussions

### GFKP-19 suppresses the Aβ_25−35_ induced cytotoxicity in neuro-2a

**Figure 1:**
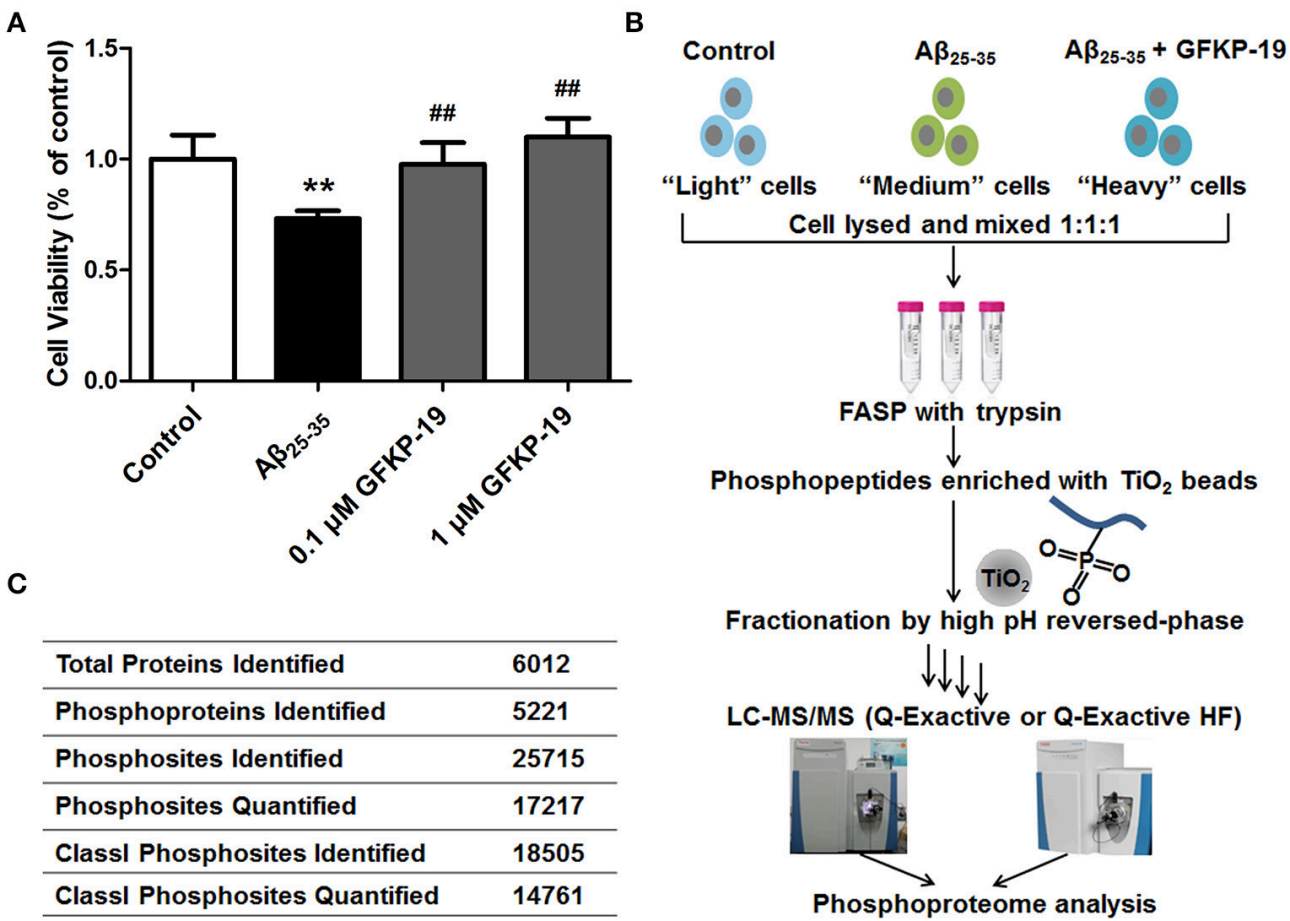
**Schematic overview of experiment. (A)** GFKP-19 can reverse the cytotoxic effect of Aβ_25−35_ in neuro-2a. Determination of the cell viability of Neuro-2a after treatment with Aβ_25−35_ in the absence or presence of GFKP-19 was carried out cells by MTT assay. Neuro-2a cells were exposed to different concentrations of GFKP-19 (0, 0.1, 1 μM) combined with 1 μM Aβ_25−35_ for 24 h. The cell viability was determined by MTT assay (*n* = 5, ^**^P < 0.01 vs. control, ^*##*^*P* < 0.01 vs. Aβ_25−35_-treated group). **(B)** Flow diagram of the SILAC-based quantitative phosphoproteomics analysis. Neuro-2a cells cultured in light, medium and heavy SILAC-labeling media was severally treated with DMSO as control, Aβ_25−35_ (1 μM) or Aβ_25−35_ (1 μM) combined with GFKP-19 (1 μM) for 4 or 24 h. Cell lysates to be compared were mixed, trypsin digested using filter-aided sample preparation (FASP), enriched on TiO_2_ beads to obtain phosphopeptides, fractionated with high pH reversed-phase and analyzed by online LC-MS/MS. Q Exactive and Q Exactive HF were used. **(C)** Overview of the LC-MS/MS results.

To determine whether GFKP-19 can protect neuro-2a cells (a brain neuroblastoma cell line) against the cytotoxicity induced by Aβ_25−35_, neuro-2a cells were treated with 1 μM Aβ_25−35_ in the absence or presence of GFKP-19 at different doses. When treated with GFKP-19 and Aβ_25−35_, cells were pretreated with GFKP-19 for 1 h before Aβ_25−35_ was added in. According to cell viability assayed by MTT method, the applicable concentration of Aβ_25−35_ was decided according to the results of prevent experiments (Figure S1A) and previous report (Shih et al., 2011). Furthermore, we found that the treatment with Aβ_25−35_ significantly decreased cell viability as assessed, and GFKP-19 was able to reverse the change (Figure 1A and Figure S1B). Thus, the above cell viability measurements qualified the samples for further proteomic analysis.

### Quantitative and comprehensive phosphoproteome analysis in neuro-2a cells

To gain global insight into the protective mechanism of GFKP-19, we combined the quantification method SILAC, high-pH reversed phase HPLC for fractionation, TiO_2_ chromatography for phosphopeptide enrichment and high accuracy mass spectrometry characterization (Figure 1B and Methods). The study was carried out on neuro-2a cells, which were cultured in light, medium and heavy SILAC-labeling media paralleling three different treatment conditions selected based on the cytotoxicity experiments (DMSO, 1 μM Aβ_25−35_, 1 μM Aβ_25−35_ + 1 μM GFKP-19). After treated with drugs for 4 or 24 h, cells were lysed and the proteins were mixed at the ratio of 1:1:1 and digested by trypsin with the method FASP. Subsequently, peptides were incubated with TiO_2_ and phosphopeptides were enriched. Next, phosphopeptides of each sample were separated into 14 fractions to decrease the complexity of the samples. The light, medium and heavy stable isotope labeled phosphopeptides were detected with highly accurate LC-MS/MS which incorporates a nanoLC system and a Q Exactive (QE) or a Q Exactive HF (HF). QE is a high-performance quadrupole Orbitrap mass spectrometer (Michalski et al., 2011) and HF denotes the improved version of QE which feature several innovations (Scheltema et al., 2014). Mass spectrometric data generated from QE and HF of three experiment groups was searched in MaxQuant using rigorous criteria. Totally, 25715 phosphosites were identified, corresponding to 5221 phosphoproteins. Among them, 18505 Class I phosphosites (of which localization probability is at least 0.75 and the score difference is 5 or higher, Table S1) (Pan et al., 2009) were identified and 14761 of them (that is, 80%) were quantified (Figure 1C).

**Figure 2:**
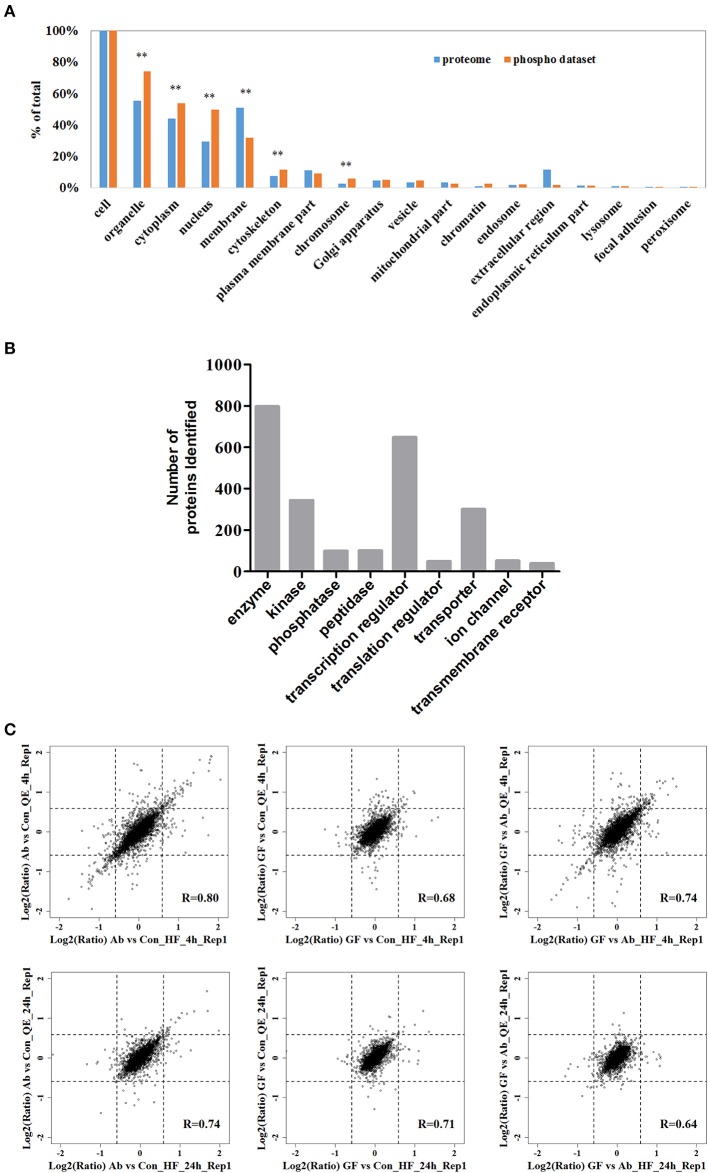
**The fractionated phosphopeptides were analyzed by an EASY n-LC 1000 system directly connected to a Q Exactive (QE) or a Q Exactive HF (HF). (A)** Distribution of phosphoproteins identified in cells. Identified phosphorylation proteins were assigned to DAVID GO terms for cellular component. Numbers of proteins belonging to each GO term are demonstrated as percentages of the total number of proteins. GO terms significantly over- and under-represented are marked [fold enrichment ≥ 1.4 or ≤ 0.67, *P* ≤ 0.01 (^**^)]. **(B)** Function classification of phosphoproteins identified. The sorting was done with Ingenuity IPA and the bar plot was generated with GraphPad. **(C)** Scatter plots of the log-transformed ratios Aβ_25−35_/Control (Ab vs. Con), (Aβ_25−35_+GFKP-19)/Control (GF vs. Con), and (Aβ_25−35_+GFKP-19)/Aβ_25−35_ (GF vs. Ab) demonstrate the correlation between QE results and HF results. Black dash lines represent 1.5 FC. The correlation coefficient (R) were calculated using spearman.

Next, we analyzed the coverage of cellular functions of the deep phosphoproteome measured. To do so, we compared the Gene Ontology (GO) annotation for cellular compartment between murine genome and identified phosphoproteins in neuro-2a cells. GO cellular component analysis, using DAVID, revealed organelle, cytoplasm, nucleus, cytoskeleton, chromosome, chromatin, endosome and vesicle to be overrepresented, indicating that the functions of these parts of cells are based on significant phosphorylation. As expected, extracellular proteins in membrane, focal adhesion and extracellular region were underrepresented (Figure 2A). This is in agreement with previous reports proving low phosphorylation in the extracellular matrix (Weintz et al., 2010). The phosphoproteins identified were classified with Ingenuity IPA for protein class analysis (Figure 2B). These results suggest the phosphoproteome reflected essential biological functions, such as kinase (*N* = 344), phosphatase (*N* = 99), transcription regulators (*N* = 649) and other processes and their regulations between conditions, demonstrating the direct relevance of studying phosphoproteome in Aβ cytotoxicity.

### Analysis of global extent of phosphoproteomic regulation

We then focused on the quantified phosphoproteomic regulation. Benefiting from the fact that the experimental replicates were performed at two independent mass spectrometers and analyzed separately, we first correlated the ratios Aβ_25−35_/Control (Ab vs. Con), (Aβ_25−35_+GFKP-19)/Control (GF vs. Con) and (Aβ_25−35_+GFKP-19)/Aβ_25−35_ (GF vs. Ab) obtained from two mass spectrometer (QE and HF). We concluded three important points in our biological and experimental system. *Firstly*, the global regulation of phosphoproteome is minor between conditions, because the fold-changes (FCs) of majority (>90%) of the phosphosites remained below 1.5 FCs. Also, GF vs. Con at 4 h only have minor change suggesting that GFKP-19 has small effect on normal neuro-2a cells. This validates our experimental approach where specific, rather than broad, phosphoprotein regulations (e.g., those triggered by cell death) were captured. *Secondly*, we found the correlations of Aβ_25−35_/Control (Ab vs. Con) and (Aβ_25−35_+GFKP-19)/Control (GF vs. Con), as well as the numbers of significantly regulated phosphorylation events were both higher at 4 h compared to those of 24 h (Figure 2C). For the 4 h data of QE and HF, 5.5 and 6.3% of the phosphosites have the FC of Ab vs. Con at least 1.5; 1.3 and 2.0% of the phosphosites have the FC of GF vs. Con at least 1.5; 4.0 and 4.7% of the phosphosites have the FC of GF vs. Ab at least 1.5. For the 24 h data of QE and HF, 2.1 and 2.7% of the phosphosites have the FC of Ab vs. Con at least 1.5; 1.2 and 1.3% of the phosphosites have the FC of GF vs. Con at least 1.5; 1.0 and 1.5% of the phosphosites have the FC of GF vs. Ab at least 1.5. This means that 4 h is a good time point to inspect phosphorylation regulation, while the status at 24 h only have minor phosphoproteomic changes. This is consistent to the rapid responsive role of phosphorylation in cellular systems (Roux and Thibault, 2013). *Last but not the least*, we managed to obtain overall decent correlations between the data sets generated from the two mass spectrometers, despite of the global phosphoproteomic changes were minor. The correlation of the repeat experiments between QE and HF can be seen in Figure S2A. As expected, HF, which has the higher instrumental configuration, generated a larger data set compared to QE (Figure S2B). The biological compositions of phosphoproteome reflected by the two instruments are also very consistent. Just as an example, for the 4 h data of QE, identified phosphosites of class I sites consist of 88.3% phosphor-Ser sites, 11.2% phosphor-Thr sites, and 0.5% phosphor-Tyr sites; for the 4 h data of HF, most phosphosites of class I sites were on Ser (88.1%) and Thr (11.0%), whereas 0.8% phosphosites were at Tyr (Figure S2C). The high inter-instrument correlation essentially provides a benchmark for our quantification data, suggesting the phosphoproteomic regulations, although they were mostly of <1.5 FCs, are not due to experimental noise. To summarize, our integrative method captured the phosphoproteomic snapshots at two time points between three conditions (Ab vs. Con, GF vs. Con, and GF vs. Ab), revealing phosphoproteome regulations are globally of a small extent but biologically specific.

### Significant protein phosphorylation regulated by Aβ_25−35_ and GFKP-19 and their patterns

**Figure 3:**
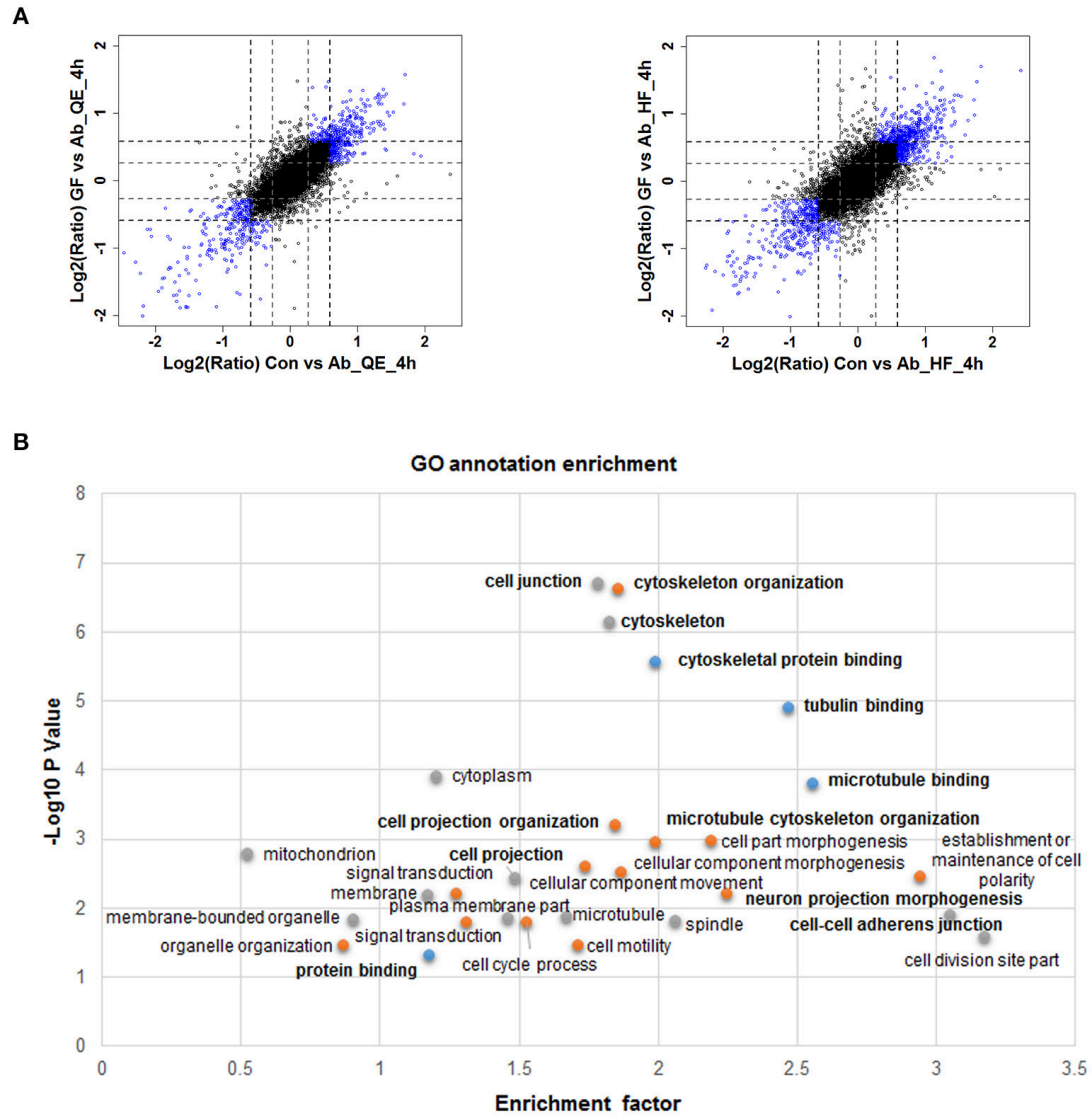
**Quantitative proteomics reveals protein phosphorylation regulated by Aβ_**25−35**_ and GFKP-19. (A)** Screening changed phosphosites with the standard: both Con vs. Ab and GF vs. Ab undergo FC at least 1.5 or one of them experience more than 1.2 FC and another at least 1.5 FC. Phosphosites identified using QE and HF with FC meeting demand were drew as blue circles and the remaining phosphosites were drew as black circles. Black dash lines represent 1.5 FC and gray dash lines represent 1.2 FC. **(B)** GO annotation enrichment of the phosphoproteins corresponding to the changed phosphosites.

Given the relatively slow and mild effects on nerve cells of neuron protective drugs, we set a filter criteria to better understand the proteins whose phosphorylation were regulated by Aβ_25−35_ and GFKP-19. This is because we hypothesize the more significantly regulated phosphoproteome events are more closely related to Aβ cytotoxicity and the protective mechanisms of GFKP-19. The substantially changed phosphosites were screened as follows: *first*, for both Con vs. Ab and GF vs. Ab, at least one of twice the FC up than 1.5 was quantified by QE or HF; *second*, both Con vs. Ab and GF vs. Ab undergo FC at least 1.5 or one of them experience more than 1.2 FCs and another at least 1.5 FCs (Figure 3A, Table S2). These highlighted phosphosites could be critical to GFKP-19 function in response to the cell toxicity of Aβ_25−35_, and analysis with heat map show very small change in GF vs. Con and opposite change between Ab vs. Con and GF vs. Ab (Figure S3). To interrogate which cell function the filtered phosphosites participate in, GO annotation enrichment for cellular function of the phosphoproteins was done. Notably, cellular function like cytoskeleton protein binding, tubulin binding, microtubule binding, cell projection, cell junction have been enriched (Benjamini-Hochberg FDR threshold of 0.02 for all processes, Figure 3B). Tau plays a vital role in the microtubule assembly and stability. Main post-translational modification of tau is phosphorylation which results in tau aggregation in pathological situations (Avila et al., 2004). Neuron morphology is formed by cytoskeletal scaffolding. Hyperphosphorylation of tau is a premise for its aberrant aggregation and the formation of NFTs, which is a main neuropathological characteristic of AD. All of this suggests that Aβ_25−35_ and GFKP-19 treatment related to the cell function that involved in AD progression.

**Figure 4:**
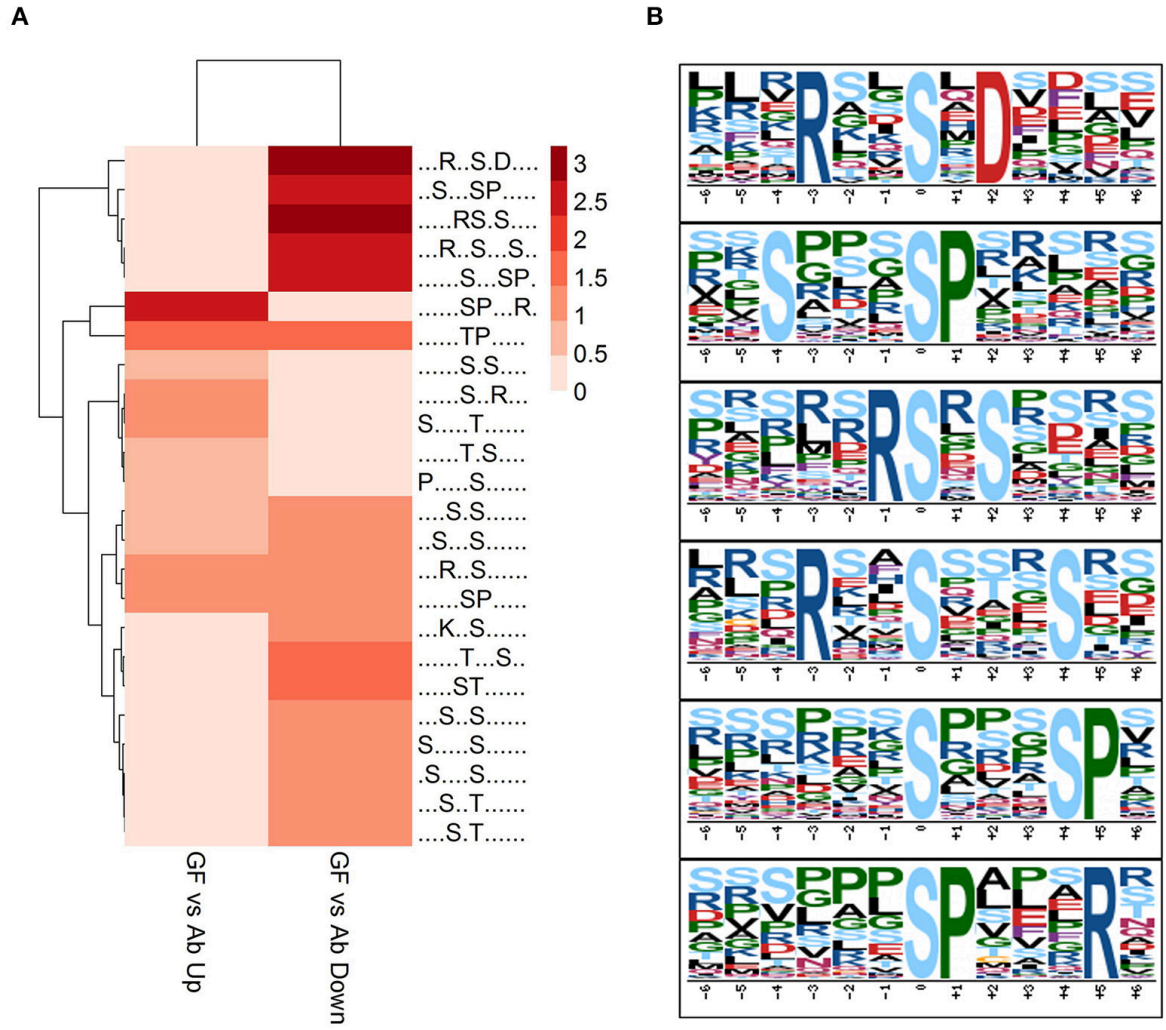
**Motif analysis. (A)** Motif analysis of phosphopeptides significantly changed in GF vs. Ab. Binary logarithm of the motif fold increase was used. Greater fold change is displayed with deeper color. **(B)** Six enriched phosphosites motifs picked up from **(A)**.

To acquire a broad understand of the phosphorylation sites and their sequence pattern significantly changed in experiment group Ab vs. Con, GF vs. Con and GF vs. Ab, we carried out motif enrichment with motif-x (Schwartz and Gygi, 2005) with a *P*-value of ≤ 1.0E-6. The analysis was done separately to the phosphopeptides that were up regulated or down regulated in Ab vs. Con, GF vs. Con and GF vs. Ab (Figure S4). More motifs were enriched for down regulated phosphosites of GF vs. Ab. Taking focus on the results of GF vs. Ab (Figure 4A), in the phosphosites up regulated we found an enrichment of proline-directed (phosphor-Ser-X-X-X-phospho-Ser-Pro), basophilic (Arg-phospho-Ser-X-phospho-Ser and Arg-X-X-phospho-Ser-X-X-X-phospho-Ser), and basophilic at left and basophilic at right (Arg-X-X-phospho-Ser-X-Asp) motifs, whereas the phosphosites down regulated were enriched for basophilic and proline-directed (phosphor-Ser-Pro-X-X-X-Arg) motif (Figure 4B). The phosphorylation of proteins with these motifs can possibly be regulated by Aβ_25−35_ and GFKP-19. Further experiments are needed to verify the functional relevance of AD for these phosphopeptide motif patterns.

### GFKP-19 potentially regulates phosphorylation of tau

How does GFKP-19 protect neuro-2a cells from the cytotoxicity of Aβ_25−35_? β-Amyloid developing SPs in AD brain can lead to the generation of superoxide radicals which react with nitric oxide (NO) producing peroxynitrite and induces cell injury subsequently. Meanwhile, NO has been suggested to be associated to the pathogenesis of AD (Law et al., 2001). NO is a free radical and can bind to superoxide anions to form peroxynitrite (Eliasson et al., 1999), causing significant oxidative stress. NO is produced by nitric oxide synthase (NOS) which has three isoforms NOS1, NOS2, and NOS3. Overexpression of all three isoforms of NOS is significantly involved in AD and the initiation of the early progression of disease (de la Monte and Wands, 2006). Phosphorylation of NOS1 at S852 activates NOS1 then increases NO production and protein phosphatase PP2B can dephosphorylate NOS1 at S852 (Foster et al., 2009). We found the phosphorylation of NOS1 at S847, which is corresponding to S852 at human NOS1, is decreased by Aβ_25−35_ treatment and rescued with GFKP-19 (Table S2). This suggests that GFKP-19 may bring down the oxidative damage and inflammation induced by NO through suppressing its synthase NOS1.

Apoptosis-stimulating protein of p53 (ASPP2; encoded by the Tp53bp2 gene) has been identified as an activator of the p53 family. ASPP2 stimulates tumor suppressor protein p53-mediated apoptotic response (Samuels-Lev et al., 2001; Bergamaschi et al., 2004). ASPP2 can be phosphorylated at S827 by the RAS/MAPK pathway which increases its binding to p53 and enhances the transcription function of p53 (Godin-Heymann et al., 2013). In our study, the phosphorylation of ASPP2 at S479 was detected to increase when treated with Aβ_25−35_ and was reversed with GFKP-19 treatment (Table S2). The site of S479 has been identified in previous report (Mertins et al., 2014), but the extract relationship between the ASPP2 protein function and this phosphosite is still unknown.

As we know, NFTs induced by hyperphosphorylation of tau protein is one of the main neuropathological characteristics of AD and has been a direction of AD treatment. Pre-clinical studies on small molecule inhibitors of primary kinases involved in tau phosphorylation to prevent hyperphosphorylation of tau have shown promise (Khlistunova et al., 2007; Pickhardt et al., 2007). According to previous study, there are six isoforms of tau in human central nervous system (CNS) ringing in size from 352 to 441 amino acids, which generated by alternative splicing (Hanger et al., 2009). Many kinases have been proved to be tau kinases in earlier investigations (Vázquez-Higuera et al., 2011; Martin et al., 2013). To identify the targets of these tau kinases, large-scale studies aimed at determining phosphosites were done, and produced much information as reference. Proline-directed protein kinases of tau target Ser and Thr preceding a Pro residue (SP/TP motif). Mitogen-activated protein kinases (MAPK), such as p38 is contained in proline-directed protein kinases (Martin et al., 2013).

**Figure 5:**
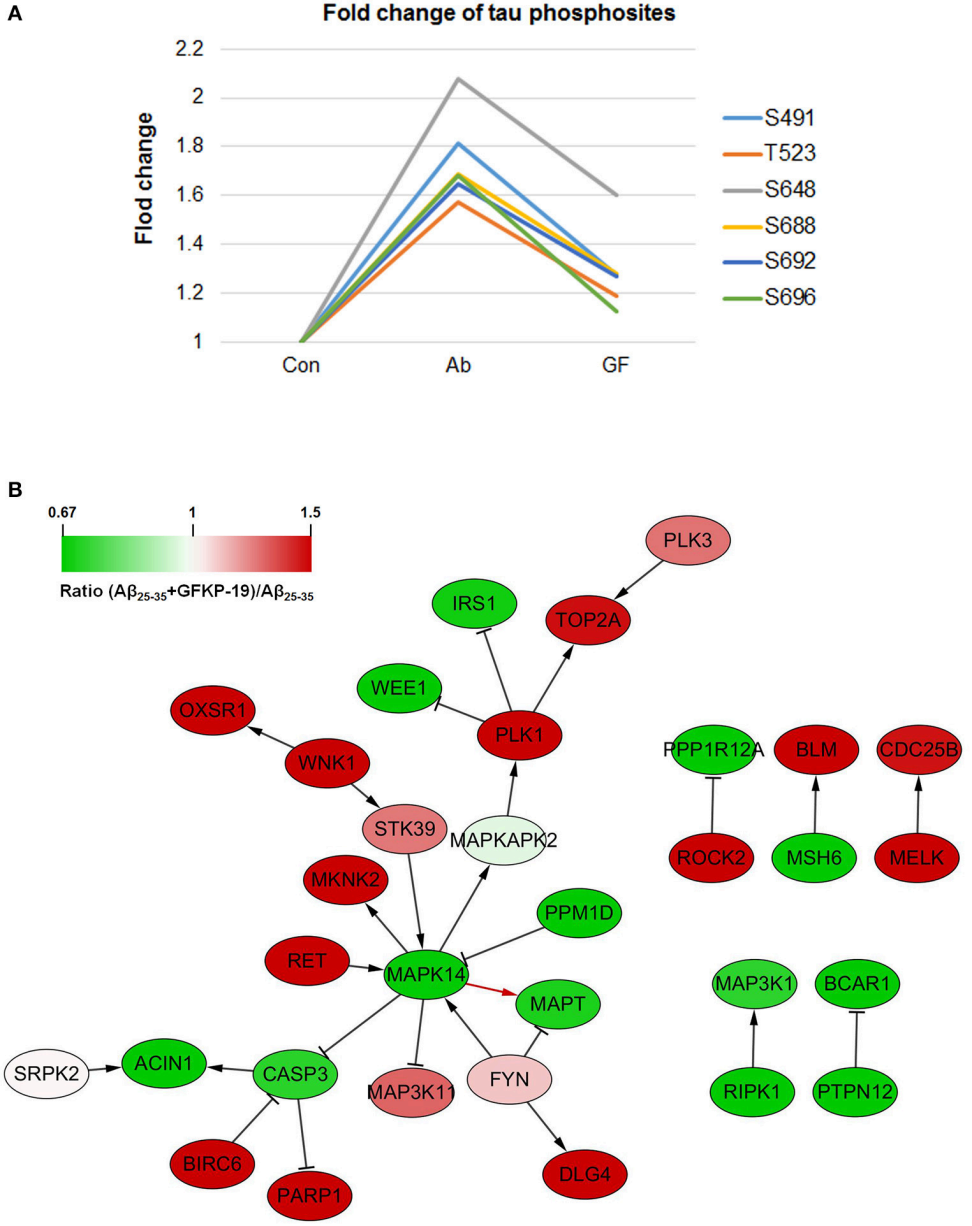
**GFKP-19 decreases the phosphorylation of tau. (A)** Fold change of tau phosphosites. The phosphorylation sites were S491, T523, S648, S688, S692, and S696 in mouse tau. The three experiment groups Control, Aβ_25−35_ and Aβ_25−35_+GFKP-19 were represented with Con, Ab and GF. **(B)** Network analysis of kinases, phosphatases and tau (MAPT). The physical interaction was provided by SIGNOR. Red represents up regulation in ratio (Aβ_25−35_+GFKP-19)/Aβ_25−35_ (GF vs. Ab), and green represents down regulation in GF vs. Ab. Interaction between MAPK14 and tau is shown with a red arrow.

In our results, 20 tau phosphosites were quantified and six of them showed up regulation after Aβ_25−35_ treated in line with previous reports that Aβ fibrils can increase tau phosphorylation (Busciglio et al., 1995), and down regulated with GFKP-19 treatment. The six phosphosites are S491, T523, S648, S688, S692, and S696 (Figure 5A, Figure S5, and Table S3), consistent to the phosphosites S199, T231, S356, S396, S400, and S404 in human tau isoform which had been reported before (Evans et al., 2000; Thornton et al., 2011; Luo et al., 2014). The phosphorylation of at S199 residue of tau is enriched in section of neurons vulnerable to neurodegeneration and is an early marker of tau pathology (Maurage et al., 2003). Phosphorylation at T231 has been assumed an initiating event for the formation of NFTs and NFTs induce neuronal cell death (Lasagna-Reeves et al., 2012). The serine 356 site located in KXGS motif promote tau disconnection from microtubules which was probably a key site refer to AD (Abraha et al., 2000; Haase et al., 2004). The phosphorylation of tau at sites Ser (396–404) was reported to represent an early event in AD (Mondragón-Rodríguez et al., 2014). These suggest that GFKP-19 can reverse Aβ_25−35_ treatment induced up regulation of tau phosphorylation.

To identify if GFKP-19 regulates the phosphorylation of tau through its kinase or phosphatases, interaction network was produced with physical interaction prediction through SIGNOR (Figure 5B). Protein phosphatase PP1 and PP2B are phosphatases of tau dephosphorylating tau at S199, S202, T205, T212, S214, S235, S262, S396, S404, and S409 and total phosphatase activity and the activity of PP2B toward tau were increased in AD brain (Liu et al., 2005). Expression product of Ppp3ca is the catalytic subunit alpha isoform of PP2B. We found the phosphorylation of Ppp3ca at S462 was up regulated after treated with Aβ_25−35_ and was down regulated by GFKP-19. The phosphorylation level of PP1 detected in our study was unchanged. Tyrosine-protein kinase FYN has been reported to phosphorylate tau at T18, but the site was not detected in our study, and the phosphorylation of FYN at Y185 was slightly up regulated with GFKP-19 treatment (Table S1), however, microtubule binding behavior of tau was not significantly altered by tyrosine phosphorylation of tau induced by FYN (Lee et al., 2004). Microtubule-associated protein/microtubule affinity-regulating kinases (MARKs) have been proved to phosphorylate the KXGS motifs of tau's repeat domain which is crucial for microtubules interactions and aggregation (Drewes et al., 1997; Biernat et al., 2002). MARK2 can phosphorylates tau at S262 and the interaction between MARK2 and tau was reported to be enhanced in AD brain section (Gu et al., 2013). MARK2 was also demonstrated to selectively phosphorylate eight serine residues in the repeat domain and C-terminal tail of tau and impact the structure and microtubule binding of tau (Schwalbe et al., 2013) and significant increase tau phosphorylation level at S396/404 and decrease total tau levels (Cavallini et al., 2013). We found that the phosphorylation of MARK2 at S499 and S533 were down regulated after treated with Aβ_25−35_ and reversed by GFKP-19, while these two sites were not previously discovered for MARK2. Mitogen-activated protein kinases (MAPK) are involved in tau hyperphosphorylation, and p38 MAPK has been reported to phosphorylate tau at S202, S205, S231, S356, and S404 (Hanger et al., 2009). MAPK1 (ERK2) can reduce phosphorylation of tau at S396 (Cavallini et al., 2013). Dual phosphorylation of MAPK1 at T183 and Y185 could be necessary for the activation of MAPK1 (Robbins et al., 1993) and we found slightly increase in the phosphorylation level of MAPK1 at these two sites induced by GFKP-19 treatment (Table S1). MAPK14 (p38α) had been reported to be a potential kinase of tau, and was related to a later onset of AD (Vázquez-Higuera et al., 2011). Phosphorylation of MAPK14 at T180 is necessary for its activation, while phosphorylation at both T180 and Y182 was more active than phosphorylation at T180 only (Zhang et al., 2008). Although we did not quantify the phosphorylation of MAPK14 at Y182, we indeed detected the change of the phosphorylation of MAPK14 at T180, a very close phosphosite (Figure S6 and Table S4). The phosphorylation level of MAPK14 at T180 was up regulated after Aβ_25−35_ treated, and down regulated with GFKP-19 treatment, suggesting that GFKP-19 may decrease tau phosphorylation through down regulating the phosphorylation level of MAPK14 at T180.

## Conclusions

Firstly, it was authenticated in the current study that cytotoxicity induced by Aβ_25−35_ in neuro-2a cells could be successfully reversed by GFKP-19 treatment within 24 h. Thus, quantitative and comprehensive phosphoproteome analysis was carried out to explore the possible protective mechanism of GFKP-19 whereby treating neuro-2a cells with three different conditions (DMSO, 1 μM Aβ_25−35_, 1 μM Aβ_25−35_ + 1 μM GFKP-19).

Secondly, we screened the significantly changed phosphosites and corresponding phosphoproteins and found that 4 h is a good time point to inspect phosphorylation regulation. Gene Ontology (GO) processes, such as cytoskeleton protein binding, cell junction, and cell projection, were closely related to Aβ_25−35_ and GFKP-19 treatment. Next, particular motifs of phosphorylation sites were discovered, with which proteins were more possibly to be regulated by Aβ_25−35_ and GFKP-19, while experiments are further required.

Thirdly, we paid special attention to the proteins involved in the hypothesis of AD. Down-regulated phosphorylation of NO synthase NOS1 at S847 after Aβ_25−35_ treatment and its reverse with GFKP-19 treatment led to a possible hypothesis that GFKP-19 may protect neuro-2a cells from the cytotoxicity of Aβ_25−35_ via suppressing NO synthase NOS1 which in turn decrease oxidative damage and inflammation induced by NO. As a crucial protein involved in AD, tau was afterwards investigated for its relevance with GFKP-19. It seems the phosphorylation level of tau increase with Aβ_25−35_ treatment and phosphorylation treatment can switch it. Meanwhile, potential tau kinase MAPK14 showed phosphorylation change at T180, which was up regulated after treated with Aβ_25−35_, and down regulated with the presence of GFKP-19. The preliminary assumption is that GFKP-19 protects neuro-2a cells from the damage of Aβ_25−35_ through decreasing tau phosphorylation which may be realized by down regulation of the phosphorylation level of MAPK14 at T180. We are currently working on generating alternative hypothesis using our data set.

In general, our three-channel SILAC strategy labeled three statuses, i.e., the control, the modeled disease, and the drug protected status, which is further combined with deep phosphoproteomic analysis enabled by extensive peptide-level fractionation and high-resolution mass spectrometry, providing a general framework to efficiently reveal the protective mechanisms of potential drugs. This strategy captures even subtle regulation events with a decent technical reproducibility and should be valuable for future similar studies.

## Author contributions

The manuscript was written by CY, YZ, YL, and HZ. CY, YL, and HZ designed the experiments. JZ, YL, and HZ supervised the experiments and revised the manuscript. CY performed the experiments with the assistance of XC and RX. CY and JG performed the statistical data analysis. All authors critically reviewed content and approved final version for publication.

### Conflict of interest statement

The authors declare that the research was conducted in the absence of any commercial or financial relationships that could be construed as a potential conflict of interest.

## Abbreviations

AD: Alzheimer's disease
SILAC: stable isotope labeling with amino acids in cell culture
Aβ: amyloid β
NFTs: neurofibrillary tangles
MS: mass spectrometry
LC-MS/MS: liquid chromatography-tandem mass spectrometry
FC: fold change
FBS: fetal bovine serum
FASP: filiter-aided sample preparation method
HPLC: high-performance liquid chromatography
QE: Q Exactive
HF: Q Exactive HF
GO: Gene Ontology
Ab vs. Con: Aβ_25−35_/Control
GF vs. Con: (Aβ_25−35_+GFKP-19)/Control
GF vs. Ab: (Aβ_25−35_+GFKP-19)/Aβ_25−35_
NOS: nitric oxide synthase
ASPP2: apoptosis-stimulating protein of p53
PP1: protein phosphatase 1
PP2B: protein phosphatase 2B
FYN: tyrosine-protein kinase
MARK: microtubule-associated protein/microtubule affinity-regulating kinase
MAPK: mitogen-activated protein kinase.
