# Supplementary material for: Deep Phosphoproteomic Measurements Pinpointing Drug Induced Protective Mechanisms in Neuronal Cells
Source: Front Physiol. 2016 Dec 23;7:635. doi: 10.3389/fphys.2016.00635 (PMC5179568; doi:10.3389/fphys.2016.00635)
Supplement: Supplementary file 3 [file DataSheet1.PDF]

Supplementary Material

Deep Phosphoproteomic measurements pinpointing drug induced protective mechanisms in neuronal cells

Figure S1. Results of MTT assays determining the concentration of $A\beta_{25-35}$ and GFKP-19 to use, related to figure 1. (A) $A\beta_{25-35}$ injuries neuro-2a cells dependent on the concentration. (B) GFKP-19 can reverse the toxicity of $A\beta_{25-35}$ to neuro-2a cells. The cell viability was determined by MTT assay (n=5, $^{**}P < 0.01$ vs. control, $^{\#\#}P < 0.01$ vs. $A\beta_{25-35}$-treated group).

Figure S2. Correlation and complementarity of quantified phosphoproteome using QE and HF, related to figure 2. (A) Correlation of the quantified phosphoproteomic data from QE and HF. (B) Venn diagrams for the phosphosites identified by QE and HF. (C) Distribution of phosphorylated Ser, Thr, and Tyr of all identified class I phosphosites.

Figure S3. Heat map for the significantly changed phosphosites screened by figure 3A. Blue represents down regulation, and orange represents up regulation.

Figure S4. Motif enrichment for the phosphorylation sites significantly changed in experiment group Ab vs Con, GF vs Con and GF vs Ab. Binary logarithm of the motif fold increase was used. Greater fold change is displayed with deeper color.

Figure S5. MS/MS spectrogram of six phosphopeptides with tau phosphosites S491, T523, S648, S688, S692 and S696 respectively.

Figure S6. MS/MS spectrogram of MAPK14 phosphosites. (A) MS/MS spectrogram of MAPK14 phosphopeptide with phosphosite only at T180. (B) MS/MS spectrogram of MAPK14 phosphopeptide with phosphosite only at Y182. (C) MS/MS spectrogram of MAPK14 phosphopeptide with phosphosites both at T180 and Y182.

Table S1, related to Figure 1 and Figure 2: Summary of the Phosphoproteome Data.

Table S2, related to Figure 3. Significantly Aβ and GFKP-19 modulated phosphosites.

Table S3, related to Figure 5A. Selective Tau phosphosites induced by $A\beta_{25-35}$ and GFKP-19 treatment.

Table S4, Quantified data of MAPK14 phosphorylation sites at S2, T180 and Y180.

Figure S1

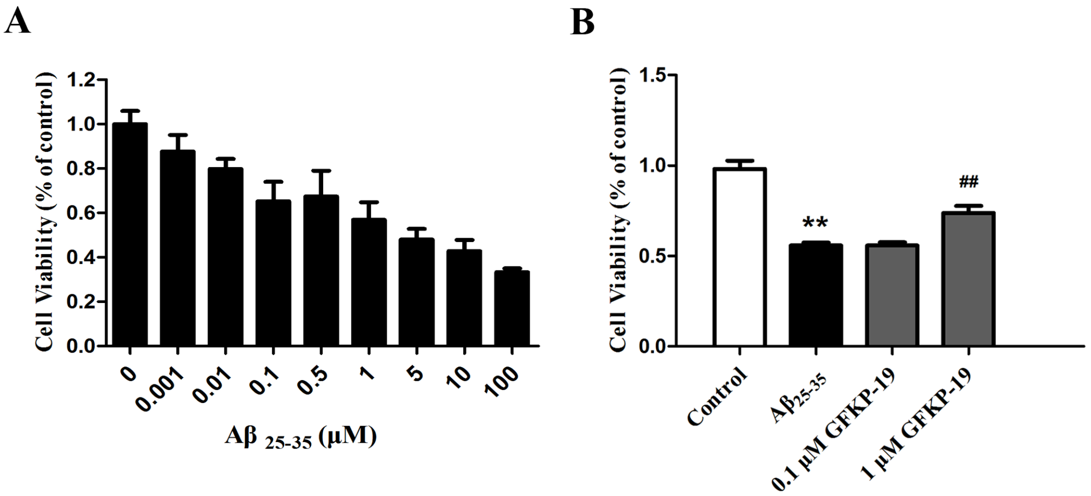
A
Cell Viability (% of control)
1.2
1.0
0.8
0.6
0.4
0.2
0.0
0
0.001
0.01
0.1
0.5
1
5
10
100
Aβ 25-35 (μM)
B
Cell Viability (% of control)
1.5
1.0
0.5
0.0
**
##
Control
Aβ25-35
0.1 μM GFKP-19
1 μM GFKP-19

Figure S2

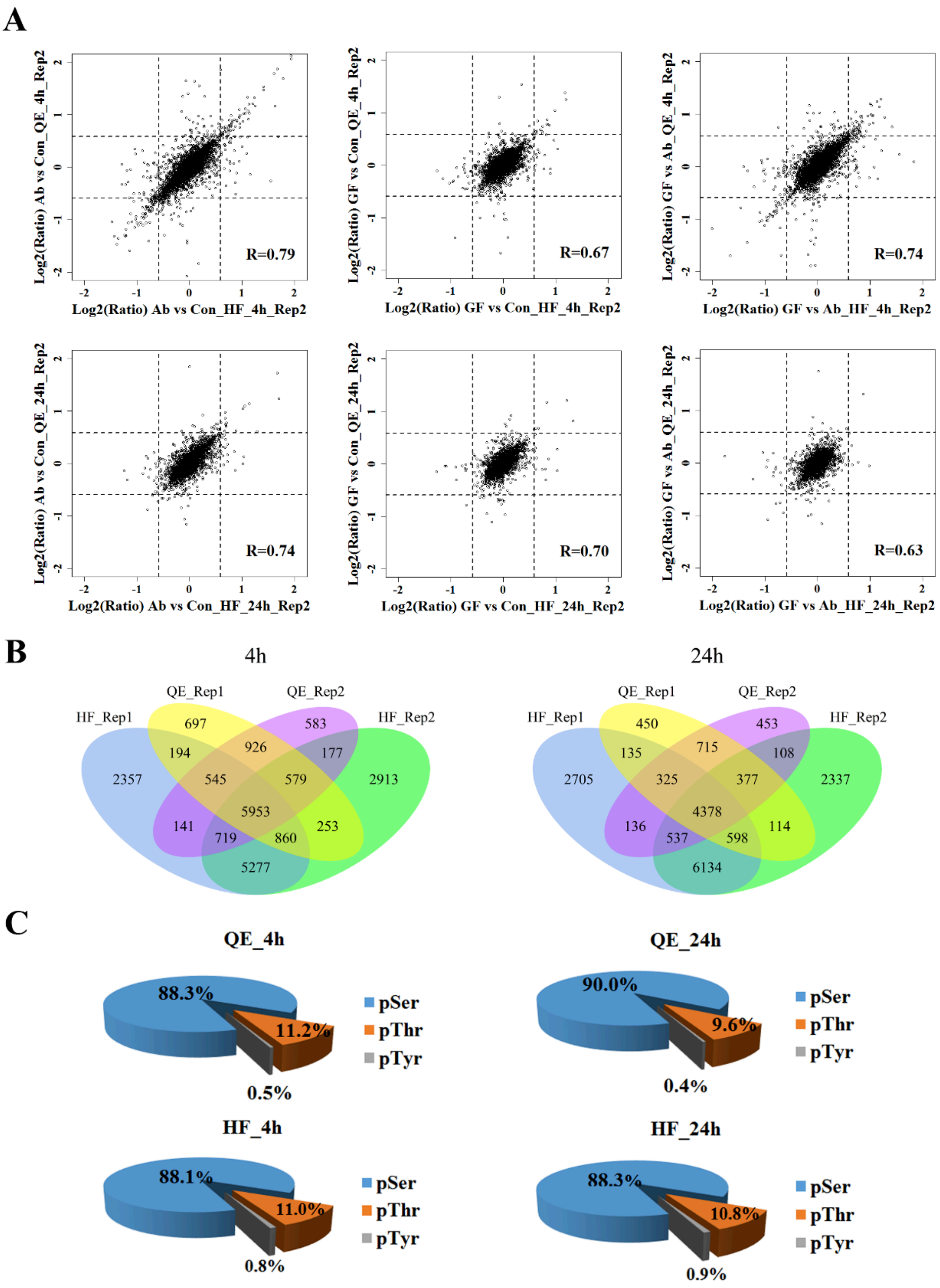
A
Log2(Ratio) Ab vs Con_QE_4h_Rep2
Log2(Ratio) Ab vs Con_HF_4h_Rep2
R=0.79
Log2(Ratio) GF vs Con_QE_4h_Rep2
Log2(Ratio) GF vs Con_HF_4h_Rep2
R=0.67
Log2(Ratio) GF vs Ab_QE_4h_Rep2
Log2(Ratio) GF vs Ab_HF_4h_Rep2
R=0.74
Log2(Ratio) Ab vs Con_QE_24h_Rep2
Log2(Ratio) Ab vs Con_HF_24h_Rep2
R=0.74
Log2(Ratio) GF vs Con_QE_24h_Rep2
Log2(Ratio) GF vs Con_HF_24h_Rep2
R=0.70
Log2(Ratio) GF vs Ab_QE_24h_Rep2
Log2(Ratio) GF vs Ab_HF_24h_Rep2
R=0.63
B
4h
HF_Rep1
QE_Rep1
QE_Rep2
HF_Rep2
697
583
194
926
177
2357
545
579
2913
5953
141
253
719
860
5277
24h
HF_Rep1
QE_Rep1
QE_Rep2
HF_Rep2
450
453
135
715
108
2705
325
377
2337
4378
136
114
537
598
6134
C
QE_4h
88.3%
11.2%
0.5%
pSer
pThr
pTyr
QE_24h
90.0%
9.6%
0.4%
pSer
pThr
pTyr
HF_4h
88.1%
11.0%
0.8%
pSer
pThr
pTyr
HF_24h
88.3%
10.8%
0.9%
pSer
pThr
pTyr

Figure S3

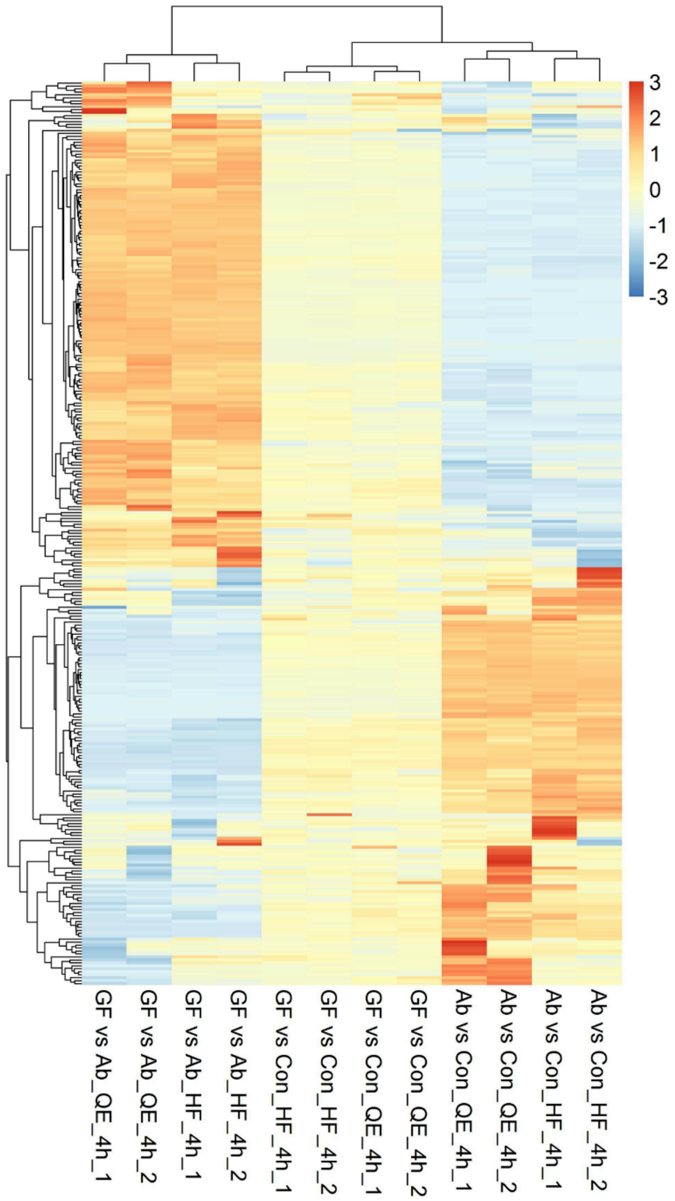
3
2
1
0
-1
-2
-3
GF vs Ab_QE_4h_1
GF vs Ab_QE_4h_2
GF vs Ab_HF_4h_1
GF vs Ab_HF_4h_2
GF vs Con_HF_4h_1
GF vs Con_HF_4h_2
GF vs Con_QE_4h_1
GF vs Con_QE_4h_2
Ab vs Con_QE_4h_1
Ab vs Con_QE_4h_2
Ab vs Con_HF_4h_1
Ab vs Con_HF_4h_2

Figure S4

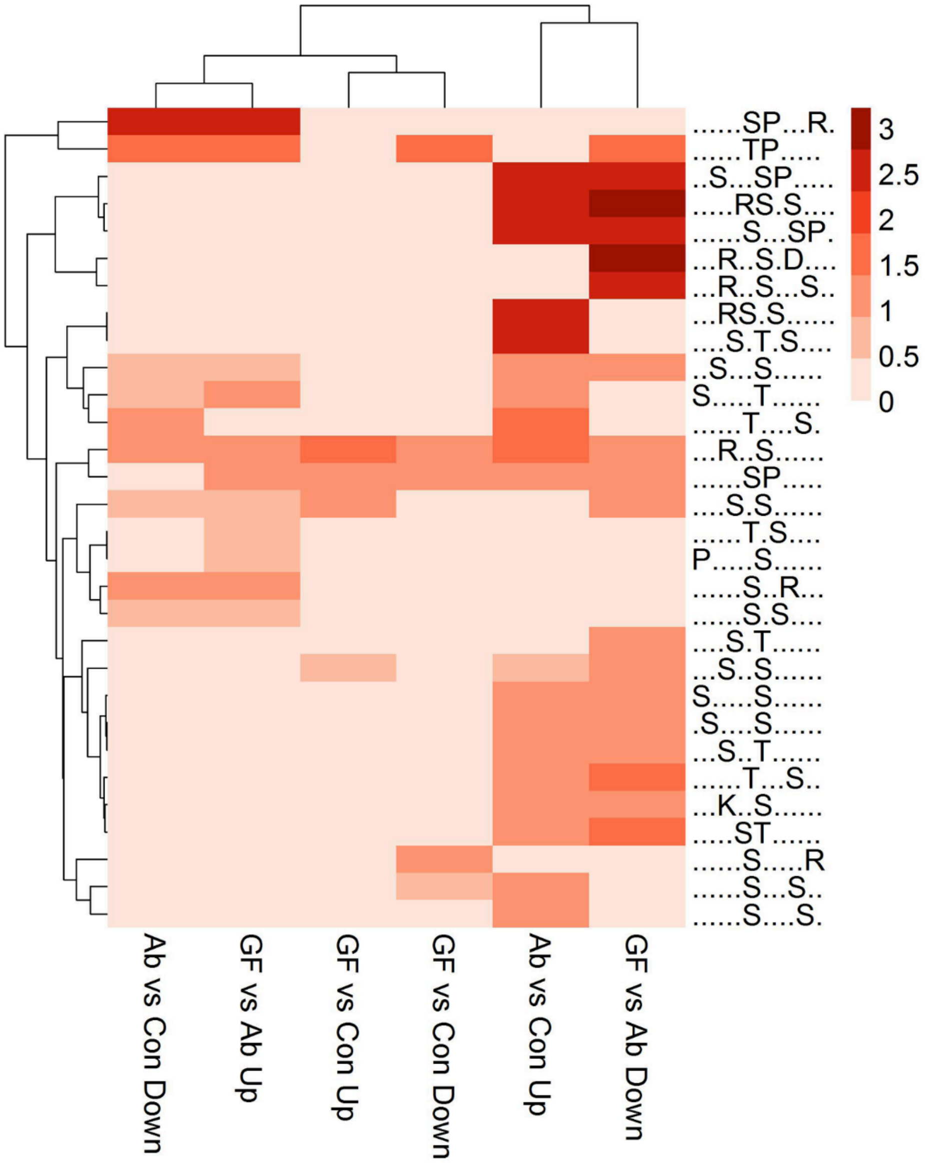
......SP...R.
......TP.....
..S...SP.....
......RS.S....
......S...SP.
...R..S.D....
...R..S...S..
...RS.S.....
....S.T.S....
..S...S......
S.....T......
......T...S.
...R..S......
......SP.....
....S.S......
......T.S....
P.....S......
......S..R...
......S.S....
....S.T......
...S..S......
S.....S......
.S....S......
...S..T......
......T...S..
...K..S......
.....ST......
......S.....R
......S...S..
......S....S.
3
2.5
2
1.5
1
0.5
0
Ab vs Con Down
GF vs Ab Up
GF vs Con Up
GF vs Con Down
Ab vs Con Up
GF vs Ab Down

Figure S5

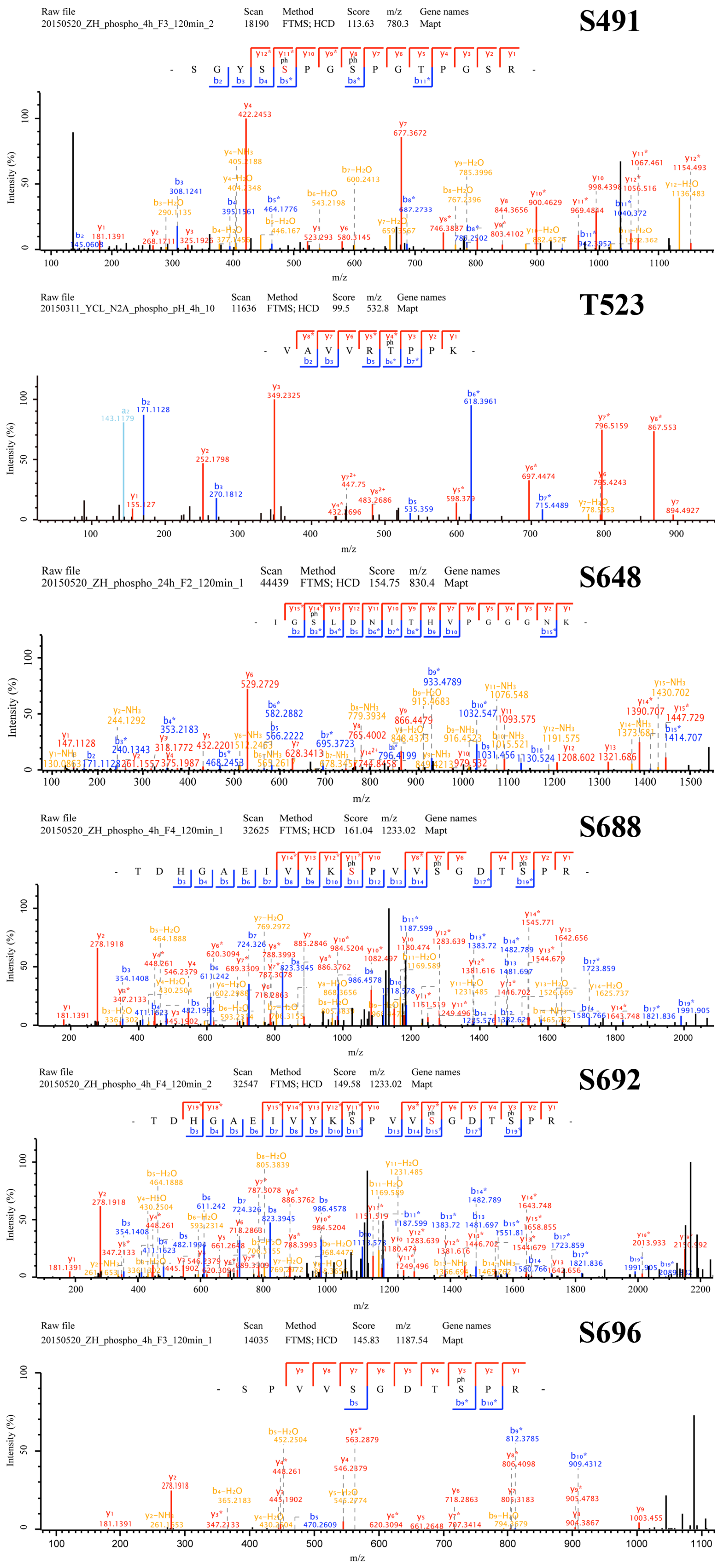

S491
Raw file 20150520_ZH_phospho_4h_F3_120min_2
Scan 18190
Method FTMS; HCD
Score 113.63
m/z 780.3
Gene names Mapt
T523
Raw file 20150311_YCL_N2A_phospho_pH_4h_10
Scan 11636
Method FTMS; HCD
Score 99.5
m/z 532.8
Gene names Mapt
S648
Raw file 20150520_ZH_phospho_24h_F2_120min_1
Scan 44439
Method FTMS; HCD
Score 154.75
m/z 830.4
Gene names Mapt
S688
Raw file 20150520_ZH_phospho_4h_F4_120min_1
Scan 32625
Method FTMS; HCD
Score 161.04
m/z 1233.02
Gene names Mapt
S692
Raw file 20150520_ZH_phospho_4h_F4_120min_2
Scan 32547
Method FTMS; HCD
Score 149.58
m/z 1233.02
Gene names Mapt
S696
Raw file 20150520_ZH_phospho_4h_F3_120min_1
Scan 14035
Method FTMS; HCD
Score 145.83
m/z 1187.54
Gene names Mapt
Intensity (%)
m/z

Figure S6

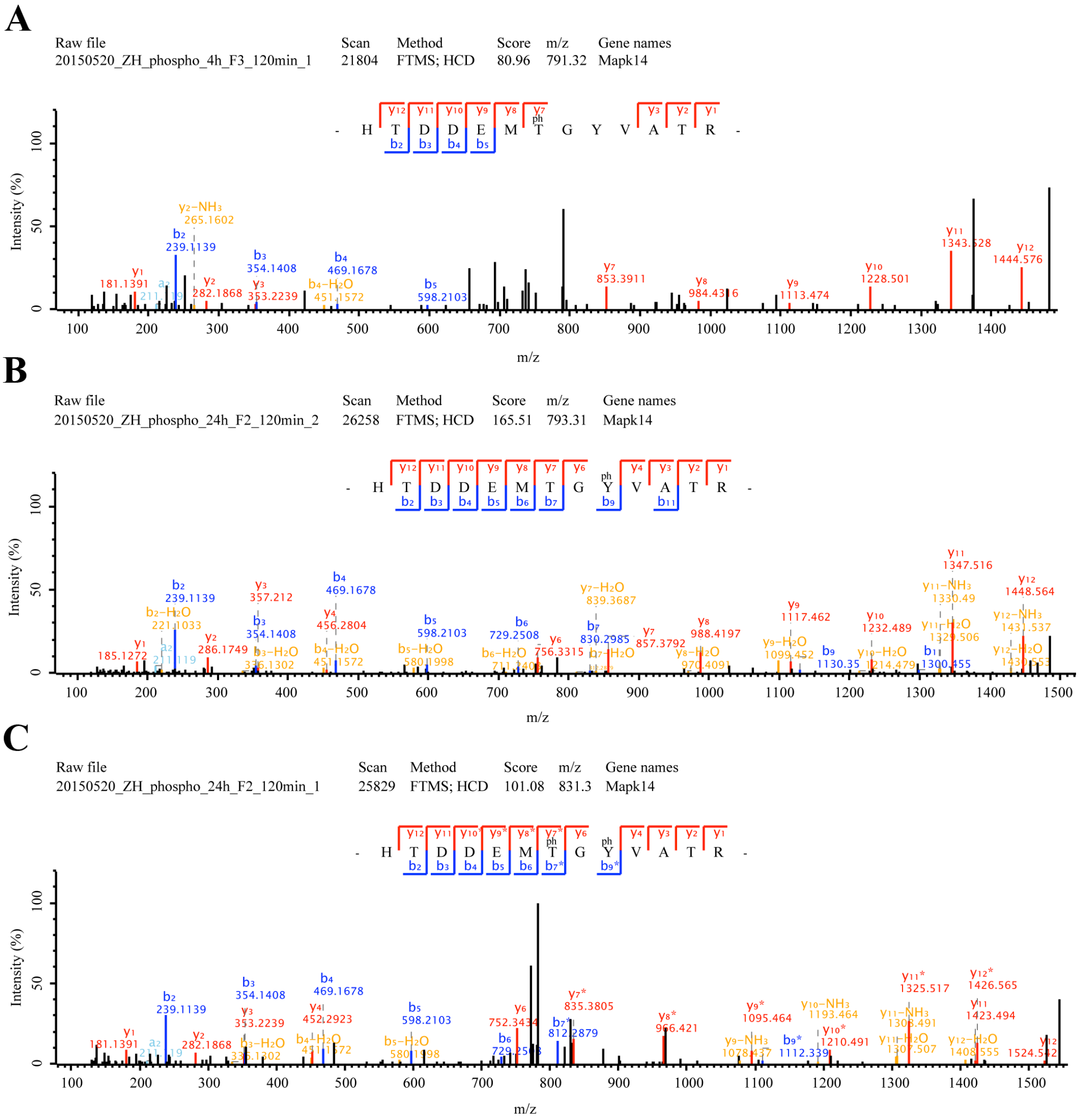
A
Raw file 20150520_ZH_phospho_4h_F3_120min_1
Scan 21804
Method FTMS; HCD
Score 80.96
m/z 791.32
Gene names Mapk14
- H T D D E M T G Y V A T R -
Intensity (%)
m/z
B
Raw file 20150520_ZH_phospho_24h_F2_120min_2
Scan 26258
Method FTMS; HCD
Score 165.51
m/z 793.31
Gene names Mapk14
- H T D D E M T G Y V A T R -
Intensity (%)
m/z
C
Raw file 20150520_ZH_phospho_24h_F2_120min_1
Scan 25829
Method FTMS; HCD
Score 101.08
m/z 831.3
Gene names Mapk14
- H T D D E M T G Y V A T R -
Intensity (%)
m/z

Table S3, related to Figure 5A. Selective Tau phosphosites induced by A$\beta_{25\text{-}35}$ and GFKP-19 treatment.

Protein	Site	Sequence window	QE_4h			HF_4h		
			Ab vs Con	GF vs Con	GF vs Ab	Ab vs Con	GF vs Con	GF vs Ab
Tau	S491	ERGEPPKSGERSGYSS PGSPGTPGSRSRTPS	1.54	1.19	0.78	1.68	1.12	0.69
Tau	T523	PTPPTREPKKVAVVRT PPKSPSASKSRLQTA	2.01	1.40	0.71	1.81	1.28	0.71
Tau	S648	SEKLDFKDRVQSKIGS LDNITHVPGGGNKKI	1.44	1.17	0.82	1.65	1.27	0.78
Tau	S688	NAKAKTDHGAEIVYK SPVVSGDTSPRHLSNV	1.56	1.24	0.79	1.69	1.28	0.76
Tau	S692	KTDHGAEIVYKSPVVS GDTSPRHLSNVSSTG	—	—	—	2.08	1.60	0.74
Tau	S696	GAEIVYKSPVVSGDTS PRHLSNVSSTGSIDM	1.55	1.23	0.75	1.57	1.19	0.75

Table S4, Quantified data of MAPK14 phosphorylation sites at S2, T180 and Y180.

Protein	Site	Sequence window	QE_4h			HF_4h		
			Ab vs Con	GF vs Con	GF vs Ab	Ab vs Con	GF vs Con	GF vs Ab
MAPK14	S2	_______________MSQERPTFYRQELNKTI	0.95	0.92	0.98	0.89	0.88	1.00
MAPK14	T180	KILDFGLARHTDDEMTGYVATRWYRAPEIML	—	—	—	1.29	0.89	0.68
MAPK14	Y182	LDFGLARHTDDEMTGYVATRWYRAPEIMLNW	—	—	—	—	—	—
